# A Rare Case of Dulaglutide-Associated Angioedema in a Male Patient

**DOI:** 10.7759/cureus.20041

**Published:** 2021-11-30

**Authors:** Nikolaos Karakousis, Nikolaos A Kostakopoulos, Vasiliki E Georgakopoulou, Elisavet E Pyrgioti, Petros N Georgakopoulos

**Affiliations:** 1 Department of Physiology, Medical School of National and Kapodistrian University of Athens, Athens, GRC; 2 Department of Gastroenterology, Medical School of National and Kapodistrian University of Athens, General Hospital of Athens "Laiko", Athens, GRC; 3 First Department of Urology, Metropolitan General Hospital, Athens, GRC; 4 Pulmonology Department, Laiko General Hospital, Athens, GRC; 5 First Pulmonology Department, Sismanogleio Hospital, Athens, GRC; 6 Department of Pharmacy, National and Kapodistrian University of Athens, Athens, GRC; 7 Internal Medicine Department, Primary Health Care Corporation, Athens, GRC

**Keywords:** swelling, angioedema, type 2 diabetes, dulaglutide, glucagon-like peptide-1 receptor agonist

## Abstract

Dulaglutide is an injectable glucagon-like peptide-1 receptor agonist approved for the treatment of adults with type 2 diabetes. Angioedema is defined as self-limiting edema, localized in the deeper layers of the skin and mucosa. Angioedema can be hereditary or acquired which can be allergic due to reactions to foods, insect bites and stings, and latex, drug-induced, caused by physical stimuli and associated with lupus erythematosus and hypereosinophilia. Angioedema represents a rare adverse event of glucagon-like peptide-1 receptor agonists. The only glucagon-like peptide-1 receptor agonist that has been mentioned to induce angioedema in literature is exenatide. We report the first case of dulaglutide-associated angioedema in a 72-year-old male in order to point out to the clinicians this potential rare side effect of this drug and its clinical significance.

## Introduction

Dulaglutide is a subcutaneous injectable glucagon-like peptide-1 (GLP-1) receptor agonist that is approved for the treatment of adults with type 2 diabetes (T2D), as monotherapy or add-on therapy to other antihyperglycemic agents, including oral antihyperglycemic drugs and insulin [1]. It is effective and well-tolerated in adults with inadequately controlled T2D and high-risk patients such as patients with obesity, elderly patients, patients with stage 3 or 4 chronic kidney disease (CKD) and/or cardiovascular (CV) disease [1].

Angioedema is self-limiting edema, which is cited in the deeper layers of the skin and mucosa and may be long-lasting [2]. Two mediators, histamine and bradykinin, are responsible for most cases of angioedema [3]. There are two kinds of angioedema, hereditary angioedema (HAE) and acquired angioedema (AAE) [4]. HAE is an autosomal dominant disease characterized by a deficiency of functional C1 esterase inhibitor (C1-INH), with type 1 being the most common [5]. On the other hand, AAE results from either consumption (type 1) or inactivation (type 2) of CI-INH [5].

AAE can be idiopathic, allergic, which is the most common and includes reactions to foods and medications (including antibiotics), insect bites and stings, and latex, drug-induced, including a commonly prescribed blood pressure drug class, angiotensin-converting enzyme (ACE) inhibitors, associated with lupus erythematosus and hypereosinophilia or caused by physical stimuli [5,6]. A helpful test that could differentiate HAE from AAE is the C1q protein. C1q protein is normal in HAE and low in AAE [4].

Herein, we present the first case of dulaglutide-associated angioedema in a 72-year-old male patient.

## Case presentation

A 72-year-old male patient with a long-term history of T2D, arterial hypertension and dyslipidemia, presented at the outpatient clinic with edema of his neck over the last three days.

**Figure 1:**
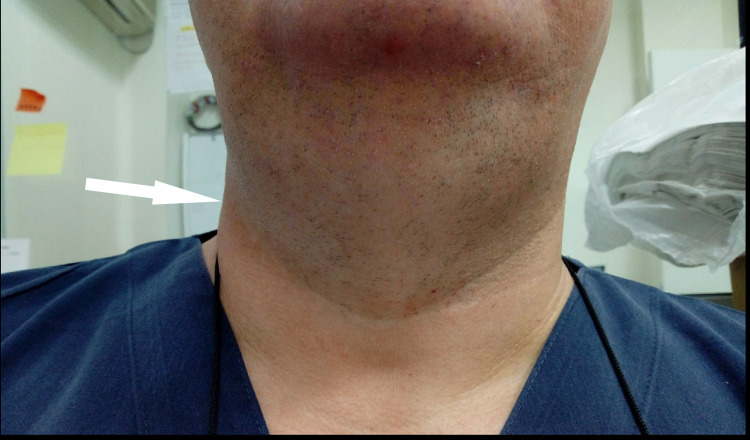
Arrow shows mildly swollen neck with moderate urticaria. No signs of infection, rashes or obvious sites of insect bite are observed.

His vital signs and chest x-ray were normal. The area of the neck was mildly swollen with moderate urticaria, but with no signs of infection, rashes or obvious sites of insect bite (Figure 1). Examination of his thyroid gland and mouth cavity did not reveal any abnormalities. The rest of the head, ears, throat, nose and eyes examination was unremarkable. Lung examination did not reveal wheezing.

The presentation was suggestive of mild angioedema of the neck. The patient reported no injuries, minor cuts or insect bites on the area. He had no previous history of medication allergy, allergic asthma, eczema or adverse reactions to other medications. He did not report any family history of angioedema. In addition, the patient denied any exposure to new foods, topical or oral creams and laundry detergents. However, the patient reported a recent change in his medication with the addition of dulaglutide, a long-acting GLP-1 receptor agonist, 0.75 mg once a week, to control his diabetes, two weeks ago. The rest of his medication was unchanged and included a combination of metformin and dapagliflozin 1,000 mg+5 mg twice a day, olmesartan 20 mg once a day and fenofibrate 145 mg once a day.

Complete blood count, including eosinophil count, and inflammatory markers were normal. Given the negative history of HAE, we did not perform a test for C1q protein. Taking into account the correlation between the addition of dulaglutide to his medication and the onset of symptoms and since angioedema is reported as a potential, extremely rare, side effect of GLP-1 medication [7], dulaglutide was discontinued and sitagliptin 100 mg once a day, was added to the already prescribed combination of metformin and dapagliflozin. No further treatment was needed and the angioedema improved significantly, the next days, after the discontinuation of dulaglutide.

Allergic angioedema is usually accompanied by pruritus and is resolved with the use of antihistamines and corticosteroids. Given the negative family history, the negative history of ACE inhibitor use, the exclusion of facial cellulitis, since the patient did not mention recent injury, fever, or facial swelling and the fact that the angioedema was resolved with no specific therapy, dulaglutide induced angioedema was the most likely diagnosis.

## Discussion

This case is the first, to the best of our knowledge, that reports the rare adverse event of dulaglutide-induced angioedema. The only case of GLP-1 receptor agonist-induced angioedema reported in the literature concerns exenatide [8]. In that case, a 67-year-old woman presented with acute-onset tongue swelling, and difficulty in breathing which occurred shortly after taking her first dose of extended-release (ER) exenatide [8].

Dulaglutide side effects include gastrointestinal complaints which are the most frequently reported, nausea, vomiting and diarrhea, hypoglycemia and elevation in serum pancreatic enzymes [9]. GLP-1 receptor agonists may contribute to antibody formation. The incidence of antibody production has been estimated at approximately 44% with exenatide, 8.6% with liraglutide, 69.8% with lixisenatide, 4% with albiglutide and 1.6% with dulaglutide. The formation of anti-exenatide antibodies was recorded to present as a diminished glycemic response rather than an anaphylactic reaction. However, the clinical relevance of these antibodies has not been clearly established [7].

Post-marketing reports demonstrate that anaphylactic reactions may present rarely with liraglutide, very rarely with exenatide, and uncommonly with lixisenatide, whilst pruritus, urticaria, and angioneurotic edema have been reported with exenatide, liraglutide, and lixisenatide. Dulaglutide was associated with systemic hypersensitivity cases in 0.5% of patients, concerning phase II and III trials [7].

Naranjo Adverse Drug Reaction Probability Scale is used to evaluate if there is a causal relationship between an adverse event and a medication. It assesses the temporal sequence of medication with the adverse event, resolution of symptoms with medication discontinuation and no alternative diagnosis possible based on medical history, clinical examination and diagnostic workup [10]. Applying Naranjo Scale to our patient, dulaglutide was a probable cause of angioedema.

The underlying mechanism for the development of drug-induced angioedema is multifactorial [11]. This mechanism for GLP-1 receptor agonists, as it is a rare adverse event, remains unclear. Although GLP-1 receptor agonists are increasingly being prescribed, this adverse effect should be taken into consideration and patients should be counseled regarding it.

## Conclusions

This is a rare case of dulaglutide-associated angioedema. This case aims to demonstrate this extremely rare potential adverse effect caused by the medical use of dulaglutide. It is of great importance for clinicians to be aware of this rare adverse event. It is also important for clinicians to recognize it early in order to confront it, depending on its severity. 
